# Herbomineral Medicine Peedanil Gold Exerts Analgesia in Neuropathy by Moderating Inflammatory Response and TRP Channel Expression in Sprague Dawley Rat Surgical Chronic Constriction Injury Model

**DOI:** 10.1155/prm/6982170

**Published:** 2025-09-10

**Authors:** Acharya Balkrishna, Shadrak Karumuri, Sandeep Sinha, Rani Singh, Rishabh Dev, Swati Haldar, Anurag Varshney

**Affiliations:** ^1^Drug Discovery and Development Division, Patanjali Research Institute, Haridwar, Uttarakhand, India; ^2^Department of Allied and Applied Sciences, University of Patanjali, Haridwar, Uttarakhand, India; ^3^Vedic Acharya Samaj Foundation, Inc. NFP, Groveland, Florida, USA; ^4^Patanjali Yog Peeth (UK) Trust, Glasgow, UK; ^5^Special Centre for Systems Medicine, Jawaharlal Nehru University, New Delhi, India

**Keywords:** allodynia, chronic constriction injury (CCI) model, hyperalgesia, p38 MAP kinase, pain receptors, Peedanil Gold

## Abstract

**Background:** Chronic neuropathic pain, a debilitating health condition, significantly deteriorates the quality of life. Available treatment options, mostly focusing on pain management, do not target underlying cause, resulting in suboptimal and temporary outcomes. Therefore, therapeutic targeting of the cause of neuropathic pain is necessary. This study was undertaken to assess the antineuropathic potential of the herbomineral formulation, Peedanil Gold (PN-G), which has earlier been proven effective against osteoarthritis and associated inflammatory pathophysiology.

**Methods:** Unilateral sciatic nerve chronic constriction injury (CCI) rat model was used to assess the analgesic and anti-inflammatory potential of PN-G in managing chronic neuropathy and associated pain hypersensitivities, by monitoring cold and tactile allodynia and thermal hyperalgesia. The mRNA expression levels of various pain receptors, TRPV1, TRPV4, TRPA1, and TRPM8, and inflammatory factors, p38 MAP kinase and interleukin-6 receptor (IL-6R), were evaluated through RT-qPCR.

**Results:** Compared to untreated study animals with CCI, PN-G treatment significantly alleviated pain hypersensitivities for cold and tactile allodynia and thermal hyperalgesia to an extent comparable to that of the reference drug, gabapentin. PN-G treatment also significantly reduced the mRNA levels of pain receptors in dorsal root ganglia, implicating a strong modulation of the pain perception. Additionally, PN-G-treated animals also exhibited noticeably reduced expressions of p38 MAP kinase and IL-6R, the crucial factors in the neuropathy-associated inflammation.

**Conclusions:** Altogether, the outcomes from the current study prove that PN-G is an effective antineuropathic agent with potential to manage pain as well as eliminate the underlying cause of neuroinflammation behind the chronicity of neuropathic pain.

## 1. Introduction

Somatosensory system is susceptible to various pathological conditions, such as metabolic disorders (peripheral diabetic neuropathy), chemotherapy-induced peripheral neuropathies, autoimmune disorders affecting the central nervous system (multiple sclerosis and Guillain–Barre syndrome), neuroinflammation, hereditary neuropathies, channelopathies, neuropathies associated with viral infections (postherpetic neuralgia, HIV, and leprosy), and traumatic damage to the nervous system (spinal cord injury [SCI] and amputation). These pathophysiological conditions ultimately lead to chronic neuropathic pain [1] with a moderate 3%–17% prevalence [2], but serious medical implications due to severely affected quality of life [1]. Neuropathic pain management guidelines based on outcomes of clinical trials recommend the use of gabapentinoids, tricyclic antidepressants, and selective serotonin–norepinephrine reuptake inhibitors as first-line drugs, lidocaine, capsaicin, and tramadol for second-line treatment, and strong opioids, like morphine and oxycodone, and botulinum toxin-A (BTX-A) as third-line options. Unfortunately, all these analgesic options have severe side effects, including, but not limited to, lethargy, vertigo, peripheral swelling, blurred vision, increased body weight, anticholinergic effects, QT prolongation (arrhythmia), suicide risk, urinary retention, constipation, ataxia, dry mouth, nausea, hyperhidrosis, hypertension, and seizures. Lidocaine and capsaicin which are administered topically often lead to erythema, itching, and rashes. Strong opioids are known to affect respiratory control, and neurotoxin BTX-A causes pain at the injection site [1]. Thus, noninvasive transcranial brain stimulation techniques, repetitive transcranial magnetic stimulation, and transcranial direct current stimulation are used [3, 4]. Nevertheless, the need for alternative options for chronic neuropathic pain management persists due to the lack of optimum treatment outcomes of the existing strategies. Herbal treatment options, such as derivatives of *Cannabis sativa* [1], puerarin, isolated from *Radix puerariae* [5], gastrodin, a bioactive constituent present in traditional Chinese herbal medicine [6], D-limonene, isolated from oranges and lemons, alone or in combination with β-cyclodextrin [7], and extract of *Azadirachta indica* [8], are being evaluated at preclinical and clinical levels [1]. Inflammation plays a prominent role in the chronic neuropathic pain etiology. Neuroinflammation, although initially responsible for regeneration and healing, due to chronicity leads to persistent pain beyond the resolution of original injuries and diseases behind neuropathy [9]. Therefore, it is important to identify alternative analgesic options for neuropathic pain which would be without adverse side effects and would target neuroinflammation as well.

In the current study, the analgesic and anti-inflammatory potential of Peedanil Gold (PN-G), a complex herbomineral formulation, in managing chronic neuropathy and associated pain hypersensitivity has been investigated [10, 11]. The effect of PN-G treatment on inflammation, crucial for the pathophysiology of neuropathic pain [9], and expressions of pain-sensing transient receptor potential (TRP) channels, namely, TRPV1, TRPV4, TRPA1, and TRPM8, were also investigated to gain mechanistic insight into the antineuropathic effect of PN-G.

## 2. Materials and Methods

### 2.1. Animal Welfare

The animal facility of Patanjali Research Institute is registered with the Committee for the Purpose of Control and Supervision of Experiments on Animals (CPCSEA), Department of Animal Husbandry and Dairying, Ministry of Fisheries, Animal Husbandry and Dairying, Govt. of India bearing registration no. 1964/PO/Rc/S/17/CPCSEA. The experimental protocol PRIAS/LAF/IAEC-086 was approved by the Institutional Animal Ethics Committee (IAEC), before study initiation. All the animal experimentation procedures and husbandry practices conformed to the CPCSEA and ARRIVE guidelines.

### 2.2. Animal Husbandry

42 male Sprague Dawley (SD) rats of 6–7 weeks of age (150–180 g) were procured from Hylasco Biotechnology Pvt. Ltd., Telangana, India, a Charles River Laboratory licensed animal supplier. Each cage of dimensions 421 (L)  × 290 (W)  × 190 (H) mm housed 4 animals at 22 ± 3°C and 30%–70% humidity under 12 h alternative dark light photoperiods. 10 to 15 air changes per hour were maintained throughout the in-life phase of this study. Animals were fed *ad libitum* with standard pelleted laboratory animal diet (Hylasco Biotechnology Pvt. Ltd., Telangana, India.). Reverse osmosis (Merck, Mumbai, India) treated, ultraviolet light sterilized drinking water was provided *ad libitum* in steam sterilized polypropylene bottles.

### 2.3. Randomization and Acclimatization

After the animals were received at the site they were quarantined for 1 week in the Laboratory Animal Facility, in an earmarked quarantine room. After the conclusion of quarantine, healthy animals were selected for the study. The animals were then transferred to an animal holding room, which was designated for the conduct of the study. Then, the body weights of the animals were noted using a pan balance (QUINTIX 1102-10IN; Sartorius, Germany). The animals were subsequently randomized on the basis of their body weight using microsoft randomization tables. During randomization, the animals were assigned a permanent animal number and marked on the middle point of the tail using nontoxic marker pen. Following randomization, animals were acclimatized for 7 days to the experimental area.

### 2.4. Preparation of the Formulation

The test article, PN-G (batch #APGT20005), sourced from Divya Pharmacy, Haridwar, Uttarakhand, India, was weighed using an analytical balance (QUINTIX 224-10IN; Sartorius, Germany) and subsequently transferred to a mortar. It was then triturated sufficiently to obtain a fine powder. After trituration, approximately 1 mL of 0.5% methyl cellulose (MC) solution was added to the powder and mixed through a syringe. After the test item was adequately wetted, the remaining volume of MC was added dropwise by employing a syringe, with continuous stirring, to obtain a stable suspension. All the formulations were prepared fresh, on each day of test article administration.

### 2.5. Animal Experiment

**Figure 1:**
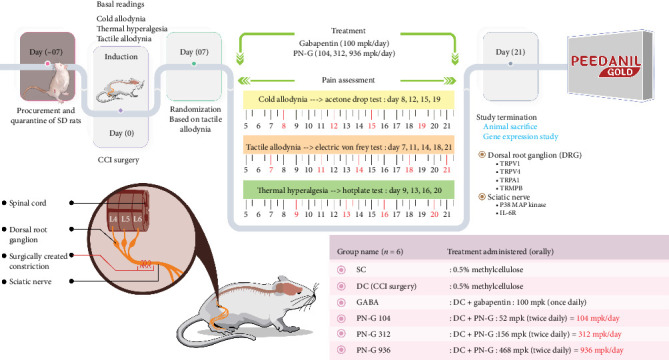
Schematic representing experimental design. The antineuropathic potential of PN-G was evaluated in chronic constricted injury model in Sprague Dawley (SD) rats. Details pertaining to study animals, their procurement, quarantine, experimental steps, doses, and dosages are provided along a timeline.

Experimental design and detailed plan are provided in Figure 1 and Table 1, respectively. Experimenters were blinded for different steps involved in the animal experiment which are described below.

#### 2.5.1. Development of Animal Model of Neuropathic Pain

Unilateral sciatic nerve chronic constriction injury (CCI) model of neuropathic pain was developed according to an earlier report by Bennett and Xie [11]. Animals allocated to groups G2–G6 were anaesthetized with an intraperitoneal injection of thiopentone sodium (50 mg/kg, Neon Laboratories, India) and surgical plane anesthesia was confirmed by absence of pedal reflexes after mechanical compression of their toes with forceps. Rats were then placed on electrically heated tables set at 37°C to prevent hypothermia during anesthesia. Then, the thigh of the right leg of the animal was subjected to hair trimming and application of 10% povidone-iodine solution (Betadine, Win Medicare, India) for sterilization. The sciatic nerve, proximal to the sciatic trifurcation, was exposed by blunt dissection of the biceps femoris muscle and freed from the adherent tissues, carefully. Subsequently, four ligatures of 4–0 chromic catgut (Ethicon, India) were placed around the sciatic nerve with a space of 1 mm between each ligature. In the animals allocated to the sham-control group, the same surgical procedures were conducted, except for the placement of ligatures. After the completion of the surgical procedures, the muscular layer and the skin were sutured, topical antibiotic dusting powder (Neosporin, Glaxo SmithKline Pharmaceuticals Ltd., India) was sprinkled, and the animals were allowed to recover from anesthesia.

#### 2.5.2. Confirmation of Development of Tactile Allodynia and Animal Randomization

Tactile allodynia measurement was conducted on day 0 (baseline) and 7 days after surgery. The animals subjected to sciatic nerve ligation were evaluated for the development of tactile allodynia by employing an electronic von Frey apparatus (Catalog No. 38450, Ugo Basile, Italy). In this procedure, rats were shifted to a noise-proof room in which the apparatus was situated and were placed in Perspex boxes with a floor made up of wire mesh. The rats were allowed to acclimatize in the boxes for 30 min. A prism, equipped with the touch stimulator transducer, facilitated the precise location of the stimulation area. Next, the metallic tip attached to the transducer was applied perpendicular to the central portion of the plantar surface of the hind paw. The pressure was progressively increased and the response to increasing pressure was defined by the paw withdrawal. The instrument automatically recorded the force after the withdrawal of the paw. To avoid damage to the plantar tissues, the cutoff value of the applied force was set to 60 g. The paw withdrawal threshold force was recorded thrice, and the average of the values was calculated and reported. The investigator who performed the measurements was blinded to the treatment.

Paw withdrawal thresholds were obtained from all the animals and the animals subjected to sciatic nerve ligation were again allocated to G2–G6 based on their paw withdrawal thresholds, ensuring similar mean paw withdrawal threshold values across the treatment groups.

#### 2.5.3. Compound Administration

From day 7 onwards, animals assigned to groups G1 and G2 received 0.5% MC, administered through oral route. Group G3 animals were orally administered 100 mg/kg gabapentin, *q.d.* (Intas Pharmaceuticals Ltd., India), according to an earlier report [12]. Animals allocated to groups G4–G6 received PN-G at the doses of 52, 156, and 468 mg/kg, *b.i.d.*, respectively, by oral gavage. For compound administration, an oral gavage tube fitted with a graduated syringe was utilized and the dose volume was maintained at 5 mL/kg of body weight, throughout the study duration. The doses selected for the study were derived by allometric scaling of the intended human dose [13, 14]. The therapeutic dose of PN-G in humans is 3000 mg/day. Consequently, total human dose is 3000 mg/60 kg/day (50 mg/kg/day). Rat equivalent doses (in mg/kg) were calculated by multiplying human equivalent dose (in mg/kg) by factor of 6.2 [14]. The therapeutic equivalent dose for rat was hence derived to be 310 mg/kg/day. Therefore, 312 mg/kg has been taken as the rat doses (human equivalent dose) and taken as the mid-dose for pharmacological studies. In order to match the human dosage, PN-G was administered twice daily to the study animals.

#### 2.5.4. Measurement of Tactile Allodynia

Tactile allodynia measurement was performed on days 7, 11, 14, 18, and 21 after the disease induction procedure by employing an electronic von Frey apparatus (Catalog No. 38450, Ugo Basile, Italy). The paw withdrawal values were compiled from all the animals of a group and expressed as mean ± standard error of (SEM). Additionally, the area under the paw withdrawal versus time (days) curve was also calculated for all the groups and expressed as mean ± SEM. Percentage inhibition of tactile allodynia was calculated by employing the following formula:

(1) $$\begin{array}{c} \%\text{ Inhibition of tactile allodynia}=\frac{\text{AUC DC group}-\text{AUC in Treated groups }}{\text{AUC in DC group}-\text{AUC in SC group}}\times 100. \end{array}$$

Furthermore, the % reversal of tactile allodynia was also calculated for each animal allocated to groups G3–G6, on days 11, 14, 18, and 21 by using the following formula:

(2) $$\begin{array}{c} \%\text{ reversal of tactile allodynia}=\frac{\text{Average paw withdrawal force in DC group}-\text{Paw withdrawal force in }a\text{ treated animal}}{\text{Average paw withdrawal force in DC group}-\text{Average paw withdrawal force in SC group}}\times 100. \end{array}$$

#### 2.5.5. Measurement of Thermal Hyperalgesia

Thermal hyperalgesia assessment was conducted on days 0 (baseline) and on days 9, 13, 16, and 20 by utilizing a hot plate (Catalog No. 35100, Ugo Basile, Italy). The instrument was set at 55 ± 1°C and the animals were gently placed on the preheated hot plate. The endpoint was a clear paw withdrawal latency time (measured in seconds). The paw withdrawal latency times were pooled for all the animals of a group and expressed as mean ± SEM. Further, the area under the paw withdrawal latency time versus time (days) curve was also calculated for all the groups, represented as mean ± SEM. Percentage inhibition of thermal hyperalgesia was calculated by employing the following formula:

(3) $$\begin{array}{c} \%\text{ Inhibition of Thermal Hyperalgesia}=\frac{\text{AUC DC group}-\text{AUC in Treated groups }}{\text{AUC in DC group}-\text{AUC in SC group}}\times 100. \end{array}$$

Furthermore, the % reversal of thermal hyperalgesia was also calculated for each animal allocated to groups G3–G6, on days 9, 13, 16, 20, and 23 by using the following formula:

(4) $$\begin{array}{c} \%\text{ reversal of thermal hyperalgesia}=\frac{\text{Average paw withdrawal latency in DC group}-\text{Paw withdrawal latency in }a\text{ treated animal}}{\text{Average paw withdrawal latency in DC group}-\text{Average paw withdrawal latency in SC group}}\times 100. \end{array}$$

#### 2.5.6. Assessment of Cold Allodynia

Cold allodynia was measured by the acetone drop test on days 0, 8, 12, 15, and 19, wherein 100 μL of acetone (RANKEM, India) was dispensed on the right paw of the animal and subsequently the paw lifting duration was measured (in seconds). The paw lifting duration data were compiled from all the animals of a group and expressed as mean ± SEM. Further, the area under the paw lifting duration time versus time (days) curve was also calculated for all the groups and expressed as mean ± SEM. Percentage inhibition of cold allodynia was calculated by employing the following formula:

(5) $$\begin{array}{c} \%\text{ Inhibition of Thermal Hyperalgesia}=\frac{\text{AUC DC group}-\text{AUC in Treated groups }}{\text{AUC in DC group}-\text{AUC in SC group}}\times 100. \end{array}$$

Furthermore, the % reversal of cold allodynia was also computed for each animal allocated to groups G3–G6, on days 8, 12, 15, and 19 by using the following formula:

(6) $$\begin{array}{c} \%\text{ reversal of cold allodynia}=\frac{\text{Average paw lifting duration in DC group}-\text{Paw lifting duration in }a\text{ treated animal}}{\text{Average paw lifting duration in DC group}-\text{Average paw lifting duration in SC group}}\times 100. \end{array}$$

Measurement of all pain parameters were conducted at the same time of the designated days. In fact, the order in which individual study animal was subjected to the tests was also maintained throughout.

#### 2.5.7. Animal Necropsy

On day 21, after completion of the recording of tactile allodynia, the animals were euthanized by an intraperitoneal injection of thiopentone (150 mg/kg). Subsequently, sciatic nerve and ipsilateral dorsal root ganglia were harvested from the animals. The proximal stub of the sciatic nerve and the dorsal root ganglia were flash frozen in liquid nitrogen and stored at −80°C for gene expression analysis.

### 2.6. Real-Time Quantitative PCR (RT-qPCR) for mRNA Expression in Dorsal Root Ganglia and Sciatic Nerve

Total RNA was extracted from dorsal root ganglia and sciatic nerve tissues of the study animals using RNeasy mini kit (Qiagen, cat no. 74106) according to manufacturer's instruction. 500 ng of total RNA was used to synthesize cDNA using Verso cDNA synthesis kit (Thermo Fisher Scientific; cat no. AB-1453/B). RT-qPCR was conducted using qTOWER3G (Germany) Real-Time PCR machine. Details of the primers used in this study are provided in Table 2. The glyceraldehyde 3-phosphate dehydrogenase (GAPDH) gene was used as endogenous control. 10 μL RT-qPCR reaction mixture containing 5 ng of template cDNA, 0.8 μL (200 nM) each of forward and reverse primers, and 5 μL of PowerUp SYBR Green Master Mix (Applied Biosystems; cat no. A25742) was used. The RT-qPCR reaction was carried out under following conditions: initial denaturation at 95°C for 5 min, following 40 cycles of amplification involving denaturation at 95°C for 30 s, annealing at 55°C for 30 s, and elongation at 72°C for 30 s with melt curve, 55°C–95°C (in 0.1°C increments). The obtained CT values were normalized against the corresponding GAPDH endogenous control gene to allow the comparison of samples independently of the amount of total input template. The effect on the expressions of different genes was determined using the 2^−ΔΔCT^ method.

### 2.7. Statistical Analysis

All the data for the studied parameters were compiled from each of the study groups and expressed as mean ± SEM. Statistical analysis was performed using GraphPad Prism Version 7.04 software (GraphPad Software, San Diego, CA, USA). Two-way analysis of variance (ANOVA) followed by Tukey's multiple comparison post hoc test was used to determine the statistically significant differences between the means for tactile allodynia, thermal hyperalgesia, and cold allodynia. One-way ANOVA followed by Dunnett's multiple comparison post hoc test was employed to calculate the statistical significance between the mean values for area under the curves for the evaluated neurobehavioral parameters and the relative mRNA expressions. A *p* value < 0.05 was considered to be statistically significant.

## 3. Results

### 3.1. PN-G Effectively Alleviated CCI-Associated Pain Sensations

Allodynia and hyperalgesia are crucial metrics in assessing pain severity, and their alleviation in response to treatments was assessed in the study animals with surgically developed CCI. These pain parameters were assessed by measuring cold and tactile allodynia through acetone drop test and von Frey assay, respectively, whereas, the effect on thermal hyperalgesia was measured through the hot plate method.

**Figure 2:**
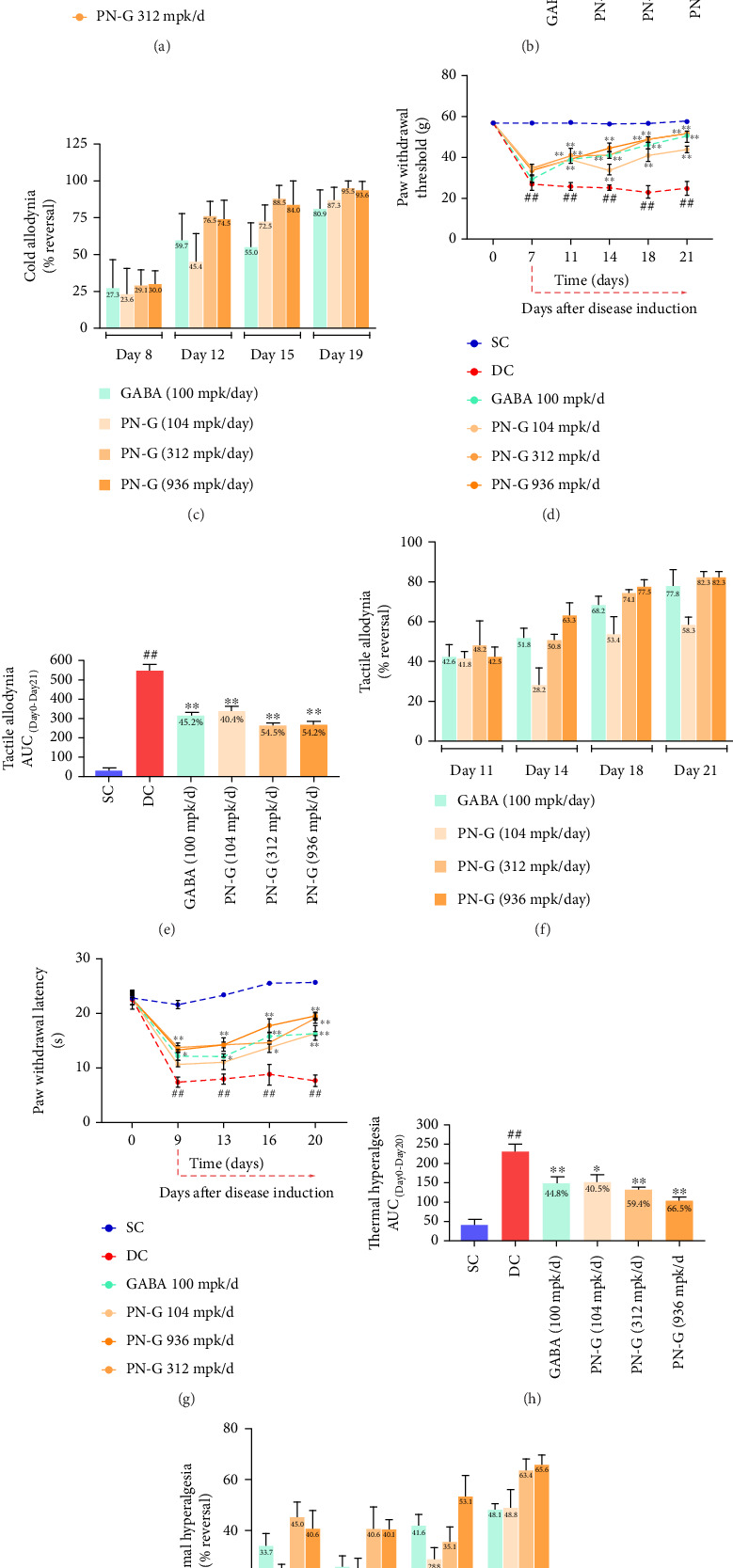
Pain hypersensitivity ameliorative effects of PN-G. (a–i) The effect of PN-G on different pain hypersensitivities, such as cold allodynia (a–c), tactile allodynia (d–f), and thermal hyperalgesia (g–i), has been depicted through monitored corresponding pain behaviors ((a) for cold allodynia, (d) for tactile allodynia, and (g) for thermal hyperalgesia). Each data point represents the mean ± SEM readings from all the animals in a designated group for the particular time point. The statistical significance of the differences between the means was determined through two-way ANOVA followed by Tukey's post hoc test and represented as ## for *p* < 0.01 when the comparison was between the SC and DC groups. The statistical significance was represented with ^∗^ and ^∗∗^, respectively, for *p* < 0.05 and 0.01, when the treatment groups were compared with DC. (b, e, h) Area under the curve (AUC) analyses of the data depicted in (a), (d), and (g) are represented as bar graphs in (b), (e), and (h), respectively. Statistical significance was determined through one-way ANOVA followed by Dunnett's post hoc test and represented with # and ## for *p* < 0.05 and 0.01, respectively, when the comparison was between SC and DC groups. For depicting the statistical significance between DC and treatment groups, ^∗^ and ^∗∗^ were used for *p* < 0.05 and 0.01, respectively. (c, f, i) Percent reversal of cold allodynia (c), tactile allodynia (f), and thermal hyperalgesia (i) in response to various treatments is depicted as grouped bars for different time intervals along the experimental tenure.

Animals subjected to CCI demonstrated cold allodynia when compared to the animals from the sham-control group (Figure 2(a)). This was evident from the longer duration of paw lifting in the animals with CCI, in response to the cold stimulus provided through application of acetone on their paws. This pain-associated behavior was not observed in sham-control animals. PN-G, administered orally at the doses of 104, 312, and 936 mpk/day, inhibited CCI-evoked cold allodynia response to acetone challenge in a dose-related manner (Figures 2(a) and 2(b)) with statistically significant changes apparent from day 12 (Figure 2(a)). With respect to the area under the curve of the paw lifting duration versus time (days) graph, PN-G significantly inhibited the cold allodynia at all the evaluated doses (Figure 2(b)). The reference drug, gabapentin administered orally at the dose of 100 mpk/day, also inhibited the observed cold allodynia; nevertheless, its effect was significant only at day 19 (Figure 2(a)). Likewise, gabapentin also depicted an overall statistically significant effect with the complete treatment regimen (Figure 2(b)). Furthermore, by day 19, PN-G at 312 and 936 mpk/day oral doses almost completely eliminated the cold allodynia, which was numerically superior to gabapentin administered at the recommended preclinical dose of 100 mpk/day (Figure 2(c)).

Similar to the occurrence of cold allodynia, CCI induced tactile allodynia, confirmed through statistically significant decrease in the paw withdrawal threshold (measured in grams) compared to the sham-control animals, 7 days after sciatic nerve ligation (Figure 2(d)). Similar decreases in the threshold were evident in all the groups subjected to surgical CCI. The statistically significant decrease in paw withdrawal threshold was maintained in the DC group throughout the study duration. Oral administration of 104, 312, and 936 mpk/day doses of PN-G efficiently reversed the ligation-induced decrease in the paw withdrawal threshold in a dose-related manner (Figure 2(d)). Significant differences between the treated groups and the DC group were evident from as early as day 11 (Figure 2(d)). Gabapentin orally administered at the dose of 100 mpk/day also significantly reversed the observed tactile allodynia, and its effect was also evident from day 11. An analysis of the AUCs, generated due to various treatments over 21 days, offered a trend of significant holistic inhibition of the tactile allodynia due to PN-G treatments at all the tested doses. Similarly, gabapentin also elicited a similar statistically significant inhibition of tactile allodynia (Figure 2(e)). Furthermore, with almost 80% reversal within 21 days (Figure 2(f)), tactile allodynia-corrective efficiency of PN-G was comparable to that of the standard drug, gabapentin.

CCI developed statistically significant thermal hyperalgesia (observed as decreased paw withdrawal latency) compared to the sham-control group, within 7 days of the sciatic nerve surgery (Figure 2(g)). Orally administered PN-G at 104, 312, and 936 mpk/day doses inhibited the CCI-induced thermal hyperalgesia in a dose-related manner (Figure 2(g)). This dose-dependent inhibition was also captured through the AUC analysis of the paw withdrawal latency versus time graph over the entire treatment regimen (Figure 2(h)). Furthermore, PN-G at orally administered doses of 312 and 936 mpk/day demonstrated approximately 65% reversal of thermal hyperalgesia, which was numerically superior to gabapentin, which offered approximately 50% reversal (Figure 2(i)).

Paw lifting duration, in case of cold allodynia and paw withdrawal threshold for tactile allodynia, returned to prelesion values at day 19 and 21, respectively. However, the paw withdrawal latency values for thermal hyperalgesia could not reach the prelesion value within the experimental tenure of 20 days, although showing an increasing trend. Taken together, PN-G treatment ameliorated different pain perceptions, and the observations were dose-dependent at low (104 mpk/day) and mid (312 mpk/day) doses. The analgesic effects exhibited by high dose (936 mpk/day) were similar to those observed at the mid dose. Therefore, the human equivalent dose (mid dose) was found to be optimal in this preclinical setup.

### 3.2. PN-G Moderates mRNA Expression of Receptors for Pain Perception in the Dorsal Root Ganglia

Targeting ion channels present in the dorsal root ganglia is considered as a better and safer option in management approach for chronic neuropathic pain [15]. Therefore, the effect of PN-G treatment on the expression of ion channels, TRPV1, TRPV4, TRPA1, and TRPM8, in the dorsal root ganglia was evaluated in the CCI rat model.

**Figure 3:**
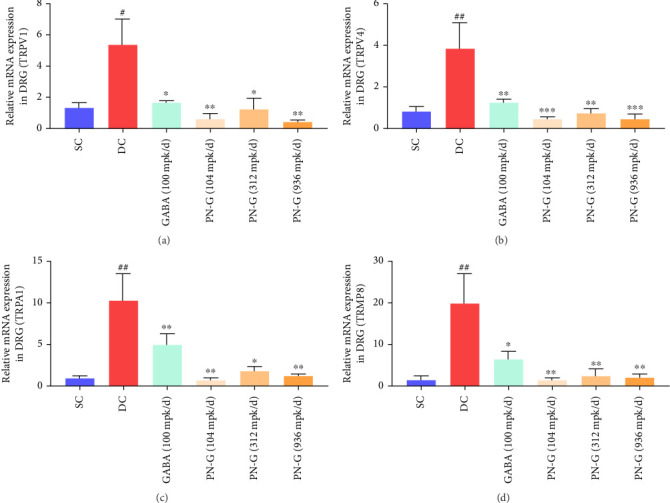
PN-G suppresses the expressions of pain receptors. (a–d) Bar graphs depicting the effect of CCI establishment and different subsequent treatments (with gabapentin and PN-G) on the mRNA expressions of pain receptors, TRPV1 (a), TRPV4 (b), TRPA1 (c), and TRPM8 (d). Statistical significance of the observed differences in the mean values is determined through one-way ANOVA followed by Dunnett's post hoc test and represented with # and ## for *p* < 0.05 and 0.01, respectively, when the comparison was between SC and DC groups. ^∗^, ^∗∗^, and ^∗∗∗^ have been used for *p* < 0.05, 0.01, and 0.001, respectively, for comparisons between DC and treatment groups.

In the DC group, the expression of TRPV1 was significantly increased, as compared to the SC group (Figure 3(a)). As expected, the reference drug, gabapentin, significantly attenuated the expression of this ion channel at the orally administered dose of 100 mpk/day. Interestingly, orally administered PN-G also significantly decreased the expression of TRPV1 at all the evaluated doses (Figure 3(a)), and the effect demonstrated by PN-G was comparable to that of gabapentin.

Similar to TRPV1, the mRNA expression of TRPV4 was also significantly increased in the animals from the DC group, as compared to their counterparts from the SC group (Figure 3(b)). PN-G significantly reduced the expression of TRPV4 at all the tested doses to an extent offered by gabapentin (Figure 3(b)).

In addition to TRPV1 and TRPV4, CCI also led to the increase in the expression of TRPA1 in the DC group, compared to the SC group (Figure 3(c)). Oral administration of PN-G significantly reduced the expression of TRPA1 at all the evaluated doses. The reference drug, gabapentin, reduced the expression of the ion channel, but its effect was less pronounced at the orally administered recommended preclinical dose of 100 mpk/day, compared to that of PN-G (Figure 3(c)). TRPM8 is yet another ion channel, the expression of which was found to be significantly increased in the dorsal root ganglia of the animals from the DC group when compared to those in the SC group (Figure 3(d)). PN-G could significantly attenuate the expression of this ion channel at all the evaluated doses, and the effect was better than that offered by the reference drug, gabapentin (Figure 3(d)).

### 3.3. PN-G Targeted Inflammatory Pathway for Chronic Neuropathy

**Figure 4:**
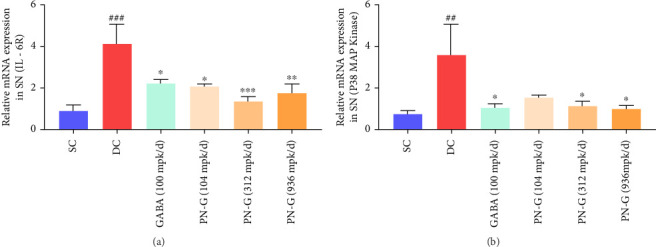
PN-G moderates the expressions of inflammatory markers. (a, b) The effect of CCI establishment and different subsequent treatments (with gabapentin and PN-G) on the mRNA expressions of IL-6R (a) and p38 MAP kinase (b). Statistical significance of the observed differences in the mean values is determined through one-way ANOVA followed by Dunnett's post hoc test and represented with ## and ### for *p* < 0.01 and 0.001, respectively, when the comparison was between SC and DC groups. ^∗^, ^∗∗^, and ^∗∗∗^ have been used for *p* < 0.05, 0.01, and 0.001, respectively, for comparisons between DC and treatment groups.

Inflammation, although initially beneficial for resolution of neural injuries, when acquires chronicity becomes an integral part of the neuropathic etiology [9, 16]. Therefore, targeting neuroinflammation could be an effective way of managing neuropathy. Microglial cells are responsible for neuroinflammation in the spinal cord, and the Schwann cells of peripheral nerves, during the degenerative phase caused by CCI, mount strong immunoreactivity [16] In the present study, mRNA levels of the neuroinflammatory markers, such as interleukin-6 receptor (IL-6R) and p38 mitogen-activated protein kinase (MAPK), were also found to be increased in the sciatic nerves of the animals from the DC group. Interestingly, like the reference drug, gabapentin, PN-G also had a suppressive effect on the mRNA expressions of these neuroinflammatory markers in the sciatic nerves that were subjected to CCI (Figure 4).

## 4. Discussion

Chronic pain, despite all efforts, continues to be a big challenge for the quality of life of the patients who have developed the pathophysiology of neuropathy. In turn, this significantly increases the disability-associated life years (DALYs) implicating a poor prognosis of this medical condition. Some of the advanced treatment options currently used are symptomatic relief or blocking pain receptors.However, they are not optimal to address the root cause of chronic neuropathic pain. The herbomineral formulation, PN-G, has earlier been shown to be very effective against osteoarthritis and associated inflammation [17]. PN-G is a complex herbomineral formulation (Table 3) [18–31] with reported anti-inflammatory effect in the murine osteoarthritic model [13]. Many of the herbal ingredients of PN-G are in common with two other polyherbal formulations, Divya-Peedantak-Kwath [32] and Divya-Peedantak-Vati [13], which have reported analgesic effects in murine chemotherapy-induced peripheral neuropathic models under preclinical conditions. The phytoconstituents of PN-G (Table 4) are known for their anti-inflammatory and nociception modulatory effects [13]. Therefore, in this study, the herbomineral formulation, PN-G, has been assessed for its chronic neuropathic pain pathology ameliorating potential using Bennett and Xie's [11] well-established unilateral sciatic nerve CCI animal model. Besides the analgesic effects, anti-inflammatory potential of PN-G has also been tested. Patients with neuropathic pain frequently experience allodynia (pain caused by a stimulus that typically does not cause pain) and hyperalgesia (increased pain from a stimulus that typically causes pain). Both are present in a variety of central and peripheral pain syndromes and impact 15%–50% of neuropathic pain sufferers. Allodynia and hyperalgesia are categorized according to the sensory modalities (touch, pressure, pinprick, cold, and heat) employed to trigger the sensation. The genesis and maintenance of these reactions are influenced by peripheral sensitization and maladaptive central alterations, with distinct processes in various subtypes of allodynia and hyperalgesia [33]. Therefore, allodynia and hyperalgesia are crucial metrics in assessing pain severity and their alleviation in response to treatments. Hence, study animals with surgically developed CCI in this study were assessed for cold and tactile allodynia and thermal hyperalgesia, according to published protocols [34–37]. PN-G assuaged thermal hyperalgesia more efficiently than gabapentin. Ion channels are known for pain perceptions and their association is identified with chronic neuropathic pain [38], making them a major group of potential drug target for chronic pain management [39]. Therefore, the effect of PN-G treatment on the expression levels of pain-sensing TRP channels, namely, TRPV1, TRPV4, TRPA1, and TRPM8, was assessed, and PN-G was found to attenuate their expressions.

PN-G could not only effectively relieve the CCI-associated pain behaviors, such as cold and tactile allodynia and thermal hyperalgesia, but also target the inflammatory pathway in the injured sciatic nerves. PN-G treatment moderated both allodynia and hyperalgesia in the study animals with CCI neuropathic pain. In case of allodynia, the pain responses, like paw lifting duration (cold allodynia) and paw withdrawal threshold (tactile allodynia), were restored to normal. However, hypersensitivity to pain stimulus in thermal hyperalgesia, although significantly reduced by PN-G treatment, was not reduced to the normal threshold, at least within the experimental tenure. p38 MAPK pathway is well-associated with CCI-associated neuroinflammation in the spinal cord through several independent studies [16]. However, optimal moderation of pain behavior by PN-G treatment in these animals was achieved at the human equivalent dose (312 mpk/day). The current study has shown that this pathway is also activated in the sciatic nerve after CCI. PN-G treatments significantly reduced the levels of p38 MAPK and one of its downstream targets IL-6R in the injured sciatic nerves. In fact, gallic acid present in PN-G is reported to exert its anti-inflammatory effect through modulation of p38 MAPK pathway in diabetic neuropathy [40]. A recent study has reported that gallic acid alleviates neuropathic pain behaviors in CCI model of rat by targeting P2X7 receptor-induced activation of NF-κB/STAT3 signaling [41]. In paclitaxel-induced neuropathic pain model, gallic acid has been reported to have ameliorative effect by moderating TNF-α and myeloperoxidase (MPO) levels [42]. Another phytocompound present in PN-G, 5-hydroxymethylfurfural (5-HMF), is known to suppress lipopolysaccharide-induced inflammation by targeting MAPK, NF-κB, and mTOR pathways in murine macrophagic RAW 264.7 cells [43]. The anti-inflammatory response of protocatechuic acid, yet another phytoconstituent of PN-G, as reported in earlier studies is versatile and is through targeting Akt, mTOR, JNK, and p38 MAPK [44, 45]. Corilagin, also found to be present in PN-G, targets MAPK and NF-κB signaling pathways to not only exert its anti-inflammatory effect but also to offer protection against hepatotoxicity [46, 47]. Anti-inflammatory effect of ellagic acid, the major phytoconstituent of PN-G, in different contexts of inflammation has been attributed to its VEGF/VEGFR2, PI3K/Akt, MAPK, and STAT signaling pathways [48, 49]. Besides, PN-G treatment resulted in reduced expressions of different pain receptors, such as TRPV1, TRPV4, TRPA1, and TRMP8. Interestingly, ferulic acid, present in PN-G, has been earlier reported to be effective against sciatica by reducing inflammation and peripheral sensitization through targeting RhoA/p38 MAPK pathway-mediated expressions of TRPA1 and TRPV1 [39]. Cinnamic acid has been found to be effective in attenuating chemotherapy-induced neuropathic pain behaviors, like cold and mechanical hypersensitivities [50]. Guggulsterones are known for their MAPK modulatory capacities, in the context of cancer [51]; nevertheless, the same effect is likely to be functional in the context of neuropathy as well.

Although the overall outcome of this study demonstrates the antineuropathic potential of PN-G, there is certain observational diffidence. PN-G treatment exhibited dose-dependent ameliorative effect on different pain responses, like allodynia and hyperalgesia. However, such dose-dependency on the effect of PN-G treatment on the expression levels of different TRP ion channels was not observed. In case of these ion channels, all the three doses of PN-G were equally effective. A similar lack of co-relation was observed on the effect of gabapentin on the levels of TRP channels and inflammatory markers, IL-6R and p38 MAPK. Although the effects of both gabapentin and PN-G on inflammatory markers were comparable, the former could reduce the expressions of TRP channels to a lesser extent than PN-G. The current study offers the preclinical background for future in-depth pharmacological and clinical studies on PN-G as a treatment option for chronic neuropathic pain. However, this study in itself does not include the toxicological safety and toxicokinetic, pharmacokinetic, and pharmacodynamic assessments of PN-G. For obvious reasons, these studies merit independent attention and, hence, could not be really identified as shortcomings of the current study. Selective molecular targets of PN-G are considered in the current study on managing chronic neuropathic pain, leaving room for other molecular targets of this test article to be identified. Therefore, these molecular targets could be a good addition in the future studies on PN-G. Nevertheless, summarily, the observations from this study and earlier independent reports on the antineuropathic effects of the phytoconstituents of PN-G reveal that this natural formulation could target both the pain receptors and the chronic inflammation as an analgesic against chronic neuropathic pain.

## 5. Conclusion

The current study was designed to assess the antineuropathic effects of the polyherbal formulation, PN-G, in the chronic constricted injury model of rat. The study intended not only to evaluate the analgesic potential of PN-G but also decipher its probable mode of action. The outcomes of this study have demonstrated that PN-G could effectively attenuate the chronic neuropathy-mediated pain behaviors, like cold and tactile allodynia and thermal hyperalgesia, by moderating the expression levels of pain receptors, like TRPV1, TRPV4, TRPA1, and TRPM8, through targeting the p38 MAPK inflammatory pathway. This study provides a comprehensive understanding of the PN-G functionality as an antineuropathic agent, which could be leveraged for further in-depth studies to develop this natural formulation into a therapeutic option against neuropathic pain.

## Figures and Tables

**Table 1:** Detailed experimental plan.

Group	Treatment	Disease induction procedure	Dose, route, and frequency of administration	Concentration of the formulation (mg/mL)	No. of animals
G1	Sham-control (SC)	Surgical exposure of sciatic nerve	0.5% MC; *p.o*., *b.i.d.*	—	7
G2	Disease-control (DC)	Loose ligation of sciatic nerve with chromic catgut (Ethicon Catgut 4-0 surgical suture)	0.5% MC; *p.o*., *b.i.d.*	—	7
G3	Reference control-gabapentin (GABA) (100 mpk/d)		100 mg/kg, p.o., q.d. in 0.5% MC	20	7
G4	PN-G (104 mpk/d)		52 mg/kg; *p.o*., *b.i.d.* in 0.5% MC	10.4	7
G5	PN-G (312 mpk/d)		156 mg/kg; *p.o*., *b.i.d.* in 0.5% MC	31.2	7
G6	PN-G (936 mpk/d)		468 mg/kg; *p.o*., *b.i.d.* in 0.5% MC	93.6	7

Abbreviations: *b.i.d*, bis in die (twice a day); MC, methyl cellulose; *p.o*, per os; *q.d*, quaque die (once a day).

**Table 2:** Details of the primers used for gene expression analysis.

Gene name	Sequence (5′-3′)	Size (bp)
TRPV1	F-AAGGATGGAACAACGGGCTAG	137
	R-TCCTGGTAGTGAAGATGTGGG	

TRPV4	F-CTTTACTTCACCCGTGGGCT	188
	R-CAGTTGCTCTGGTCCTCGTT	

TRPA1	F-GCAGCATTTTCAGGTGCCAA	172
	R-CGCTGTCCAGGCACATCTTA	

TRPM8	F-GCTACGGACCAGCATTTCAT	172
	R-GCTTGTCAATGGGCTTCTT	

IL-6R	F-AAGCAGGTCCAGCCACAATGTAG	118
	R-CCAACTGACTTTGAGCCAACGAG	

P38 MAPK	F-ACATCGTGTGGCAGTGAAGAAG	60
	R-CTTTTGGCGTGAATGATGGA	

Abbreviations: ANOVA, analysis of variance; AUC, area under the curve; BTX-A, botulinum toxin-A; CCI, chronic constriction injury; CPCSEA, Committee for the Purpose of Control and Supervision of Experiments on Animals; DALYs, disability-associated life years; IAEC, Institutional Animal Ethics Committee; IL-6R, interleukin-6 receptor; PN-G, Peedanil-Gold; rTMS, repeated transcranial magnetic stimulation; RT-qPCR, real-time quantitative PCR; SD, Sprague Dawley; SEM, standard error of mean; SNRI, selective serotonin–norepinephrine reuptake inhibitor; TCAs, tricyclic antidepressants; tDCS, transcranial direct current stimulation.

**Table 3:** Classical composition of Peedanil-Gold (PN-G).

Name of the PN-G components (classical reference [page no.])	Identity of the component (form used)	Quantity (mg/g)	Ingredients of PN-G		
			Scientific binomial name	Sanskrit binomial name	Common English name (Hindi name)
Punarnavadi Mandoor [18] (Ayurved Sarsangrah, Edition 2010 [499])	Classical formulation (powder)	111.11	*Boerhavia diffusa* L.	Punarnavakaḥ raktakāṇḍaḥ (पुनर्नवक: रक्तकाण्ड)	Spreading hogweed (Punarnava)
			*Operculina turpethum* (L.) Silva Manso	Pīnaśalkam tāmrakali (पीनशल्कम् ताम्रकलि)	St. Thomas lidpod (Trivrita)
			*Zingiber officinale* Roscoe	Ārdrakam sitauṣṭham (आर्द्रकम् सितौष्ठम्)	Ginger (Shunthi)
			*Piper nigrum* L.	Kaṇikā maricā (कणिका मरिचा)	Black pepper (Maricha)
			*Piper longum* L.	Kaṇikā pippalī (कणिका पिप्पली)	Indian long pepper (Pippali)
			*Embelia tsjeriam-cottam* (Roem. & Schult.) A.DC.	Viḍaṅgakaḥ pītasumaḥ (विडङ्गक: पीतसुम)	False black pepper (Vidanga)
			*Cedrus deodara* (Roxb. ex D.Don) G.Don	Devadrujaḥ dṛḍhakāṣṭhaḥ (देवद्रुज: दृढकाष्ठ)	Deodar cedar (Devdaru)
			*Plumbago zeylanica* L.	Citrakaḥ sitasumaḥ (चित्रक: सितसुम)	Doctorbush (Chitraka)
			*Aucklandia costus* Falc.	Kuṣṭhakaḥ dīrghamūlaḥ (कुष्ठक: दीर्घमूल)	Costus root (Kushtha)
			*Curcuma longa* L.	Haridrakā pītāṅgā (हरिद्रका पीताङ्गा)	Turmeric (Haridra)
			*Berberis aristata* DC.	Pītadrukaḥ haridruḥ (पीतद्रुक: हरिद्रु)	Indian barberry (Daruharidra)
			*Terminalia chebula* Retz.	Vīrakaḥ harītakaḥ (वीरक: हरीतक)	Chebulic myrobalan (Haritaki)
			*Phyllanthus emblica* L.	Āmalakaḥ dhātrīphalaḥ (आमलक: धात्रीफल)	Indian gooseberry (Amalaki)
			*Terminalia bellirica* (Gaertn.) Roxb.	Vīrakaḥ akṣaḥ (वीरक: अक्ष)	Beleric myrobalan (Bibhitaki)
			*Baliospermum solanifolium* (Burm.) Suresh	Dantikā aromānupatrā (दन्तिका अरोमानुपत्रा)	Wild croton (Danti)
			*Piper retrofractum* Vahl	Kaṇikā golasapatrā (कणिका गोलसपत्रा)	Piper chilli (Chavya)
			*Holarrhena pubescens* Wall. ex G.Don	Kuṭajakaḥ yavaphalaḥ (कुटजक: यवफल)	Tellicherry bark (Indrayava)
			*Cyperus rotundus* L.	Jalamuk mustakaḥ (जलमुक् मुस्तक)	Nut grass (Musta)
			Incinerated red oxide of iron (Mandura Bhasma)		Mineral and metallic components (Calx/Bhasma)

Guggul Shuddh [19] (Bhav Prakash Nighantu, 16^th^ century, Edition 2006 [205])	Plant exudate (exudate)	462.96	*Commiphora wightii* (Arn.) Bhandari	Guggulakaḥ gaṇḍiśākhaḥ (गुग्गुलक: गण्डिशाख)	Indian bdellium-tree (Guggul)

Mukta Shukti Bhasma [20] (Ras Tarangini [11^th^ century], Edition 2004 [295–298])	Classical metallic formulation (powder)	111.11			Processed pearl oyster

Mahavat Vidhvansan Ras (Ayurved Sarsangrah, Edition 2010 [373])	Classical formulation (powder)	111.11	*Piper longum* L.	Kaṇikā pippalī (कणिका पिप्पली)	Indian long Pepper (Pippali)
			*Piper nigrum* L.	Kaṇikā maricā (कणिका मरिचा)	Black Pepper (Maricha)
			*Zingiber officinale* Roscoe	Ārdrakam sitauṣṭham (आर्द्रकम् सितौष्ठम्)	Ginger (Shunthi)
			*Eclipta prostrata* (L.) L.	Bhṛṅgarājakaḥ keśyaḥ (भृङ्गराजक: केश्य)	False daisy (Bhringraj)
			*Aucklandia costus* Falc.	Kuṣṭhakaḥ dīrghamūlaḥ (कुष्ठक: दीर्घमूल)	Costus root (Kushtha)
			*Vitex negundo* L.	Nirguṇḍikā madhupapriyā (निर्गुण्डिका मधुपप्रिया)	Chinese chaste tree (Sambhalu)
			*Calotropis procera* (Aiton) W.T.Aiton	Arkaḥ vṛntākasumaḥ (अर्क: वृन्ताकसुम)	Giant milkweed (Safed-ak)
			*Phyllanthus emblica* L.	Āmalakaḥ dhātrīphalaḥ (आमलक: धात्रीफल)	Indian gooseberry (Amalaki)
			*Plumbago zeylanica* L.	Citrakaḥ sitasumaḥ (चित्रक: सितसुम)	Doctorbush (Chitraka)
			*Citrus limon* (L.) Osbeck	Nimbukaḥ tuṇḍaphalaḥ (निम्बुक: तुण्डफल)	Lemon (Nimbu)
			*Phyllanthus emblica* L.	Āmalakaḥ dhātrīphalaḥ (आमलक: धात्रीफल)	Indian gooseberry (Amalaki)
			*Terminalia bellirica* (Gaertn.) Roxb.	Vīrakaḥ akṣaḥ (वीरक: अक्ष)	Beleric myrobalan (Bibhitaki)
			*Terminalia chebula* Retz.	Vīrakaḥ harītakaḥ (वीरक: हरीतक)	Chebulic myrobalan (Haritaki)
			Herbal processed mercury calx (Shodhit Parad)		Mineral and metallic Components (Calx/Bhasma)
			Herbal processed sulfur calx (Shodhit Gandhak)		
			Lead calx/herbally processed lead (Naga Bhasma) [21, 22]		
			Tin calx/naturally processed Tin) (Vanga Bhasma) [23]		
			Calcined Iron calx (Lauha/Loha Bhasma) [24]		
			Incinerated Copper calx (Tamra Bhasma) [25, 26]		
			Purified and processed Mica calx (Abhraka Bhasma) [27]		
			Borax calx (Tankana Bhasma)		
			Purified Aconitum Ferox calx (Shuddha Vatsanabha)		

Amvatari Ras [28] (Ayurved Sarsangrah, Edition 2010 [260])	Classical formulation (powder)	111.11	*Terminalia chebula* Retz.	Vīrakaḥ harītakaḥ (वीरक: हरीतक)	Chebulic myrobalan (Haritaki)
			*Terminalia bellirica* (Gaertn.) Roxb.	Vīrakaḥ akṣaḥ (वीरक: अक्ष)	Beleric myrobalan (Bibhitaki)
			*Phyllanthus emblica* L.	Āmalakaḥ dhātrīphalaḥ (आमलक: धात्रीफल)	Indian gooseberry (Amalaki)
			*Plumbago zeylanica* L.	Citrakaḥ sitasumaḥ (चित्रक: सितसुम)	Doctorbush (Chitraka)
			*Commiphora wightii* (Arn.) Bhandari	Guggulakaḥ gaṇḍiśākhaḥ (गुग्गुलक: गण्डिशाख)	Indian bdellium-tree (Guggul)
			*Ricinus communis* L.	Eraṇḍakaḥ pañcāṅgulaḥ (एरण्डक: पञ्चाङ्गुल)	Castor Bean (Arandi)
			*Vachellia nilotica* (L.) P.J.H.Hurter & Mabb.	Marandavṛntakam babbūlam (मरन्दवृन्तकम् बब्बूलम्)	Indian Gum (Babool)
			Herbal processed mercury (Shodhit Parad)		Mineral and metallic Components (Calx/Bhasma)
			Herbal processed sulfur (Shodhit Gandhak)		

Vrihatvat Chintamani Ras [29] (Bhaishajya Ratnavali [18^th^ century], Eighteenth Edition [543–544])	Classical herbometallic formulation (powder)	18.52	*Aloe vera* (L.) Burm.f.	Majjapatrakam aṅgārasumam (मज्जपत्रकम् अङ्गारसुमम्)	Aloe vera (Ghritkumari)
			Calcined gold calx (Swarna Bhasma) [30]		Mineral and metallic Components (Calx/Bhasma)
			Silver oxide calx (Rajat Bhasma) [30]		
			Calcined Iron calx (Lauha/Loha Bhasma) [24]		
			Purified and processed Mica calyx (Abhraka Bhasma) [27]		
			Oxide of Coral (Prawal Bhasma) [30]		
			Oxide of pearl (Mukta Bhasma) [30]		
			Herbometallic preparation from mercury, sulfur, and *Ficus benghalensis* L. (Rasasindoor) [31]		

**Table 4:** Bioactive phytochemicals present in Peedanil-Gold (PN-G), as detected on HPLC.

Name of the marker compound	Chemical structure	Quantity in PN-G (μg/mg)^∗^
Gallic acid	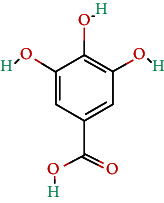	0.015

5-hydroxymethylfurfural	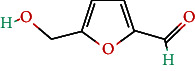	0.091

Protocatechuic acid	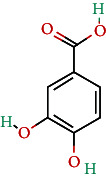	0.012

Corilagin	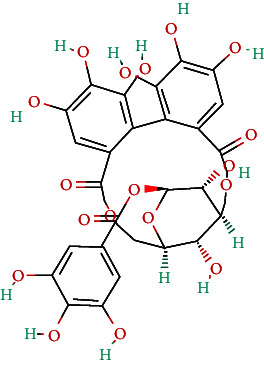	0.043

Ellagic acid	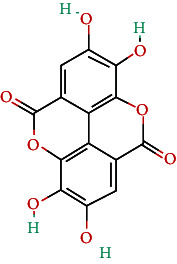	2.288

Ferulic acid	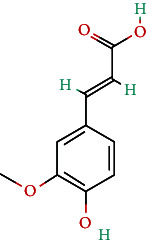	0.017

Cinnamic acid	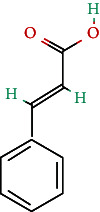	0.022

Guggulsterone E	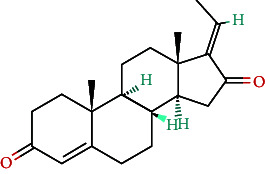	0.162

Guggulsterone Z	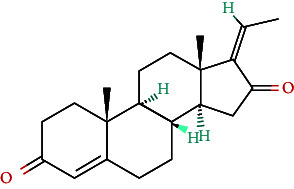	0.140

^∗^Information excerpted from reference [13].

## Data Availability

The data that support the findings of this study are included in the manuscript and are available from the corresponding author upon reasonable request.

## Supporting Information

Unilateral sciatic nerve CCI model of neuropathic pain was developed by exposing the sciatic nerve, proximally to the sciatic trifurcation by blunt dissection of the biceps femoris muscle. Subsequently, ligatures were placed around the exposed sciatic nerve. Untreated chronic constricted injury led to an increase in the DRG ion channels and inflammatory markers, which were alleviated on Peedanil-Gold treatment.
