# Supplementary material for: Herbomineral Medicine Peedanil Gold Exerts Analgesia in Neuropathy by Moderating Inflammatory Response and TRP Channel Expression in Sprague Dawley Rat Surgical Chronic Constriction Injury Model
Source: Pain Res Manag. 2025 Sep 10;2025:6982170. doi: 10.1155/prm/6982170 (PMC12443517; doi:10.1155/prm/6982170)
Supplement: Supporting Information — Additional supporting information can be found online in the Supporting Information section. [file 6982170.f1.pdf]

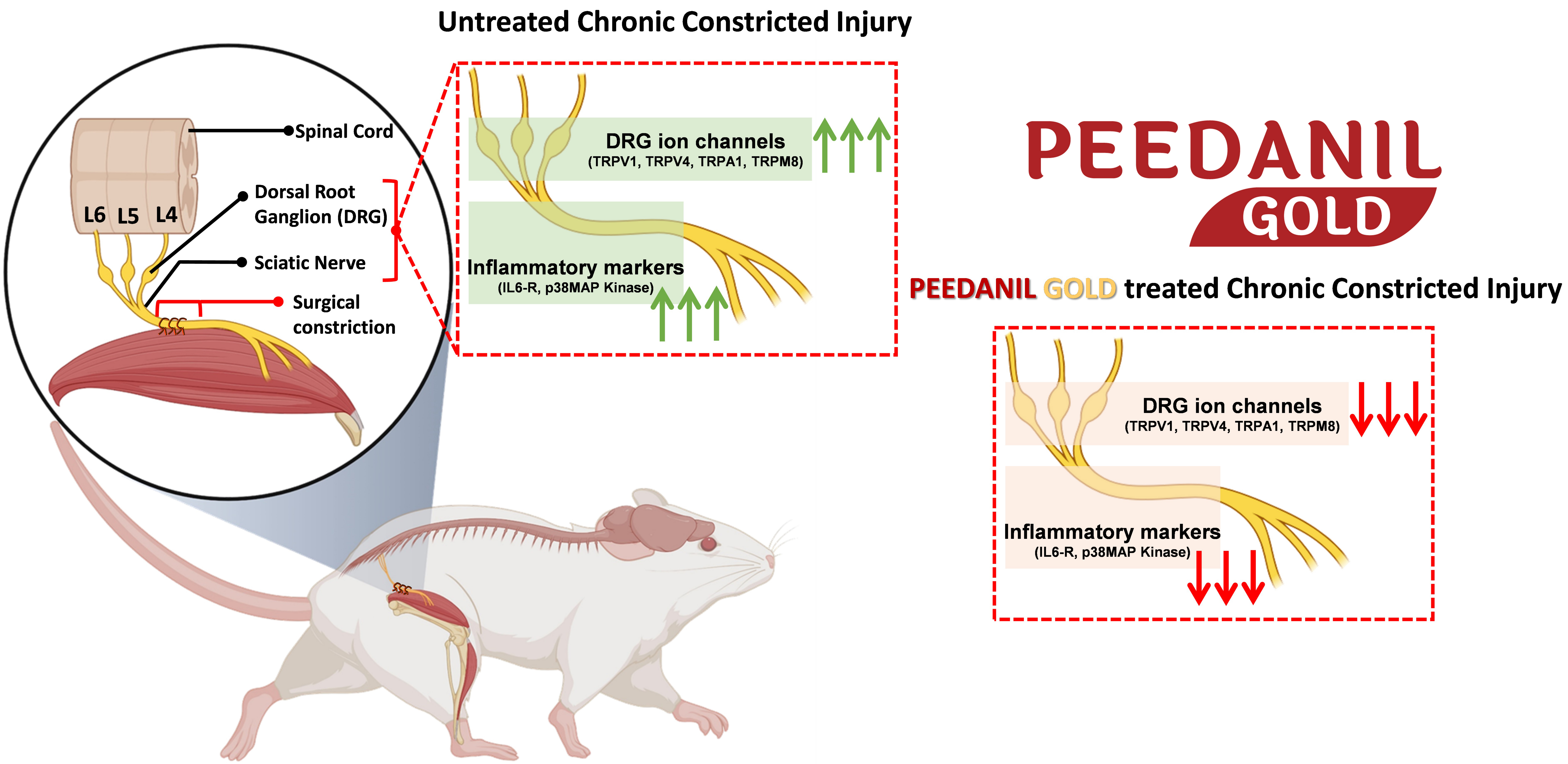

Untreated Chronic Constricted Injury
Spinal Cord
L6 L5 L4
Dorsal Root Ganglion (DRG)
Sciatic Nerve
Surgical constriction
DRG ion channels
(TRPV1, TRPV4, TRPA1, TRPM8)
Inflammatory markers
(IL6-R, p38MAP Kinase)
PEEDANIL GOLD
PEEDANIL GOLD treated Chronic Constricted Injury
DRG ion channels
(TRPV1, TRPV4, TRPA1, TRPM8)
Inflammatory markers
(IL6-R, p38MAP Kinase)
